# The cotton WRKY transcription factor (GhWRKY33) reduces transgenic Arabidopsis resistance to drought stress

**DOI:** 10.1038/s41598-018-37035-2

**Published:** 2019-01-24

**Authors:** Na-Na Wang, Shang-Wei Xu, Yun-Lue Sun, Dong Liu, Li Zhou, Yang Li, Xue-Bao Li

**Affiliations:** Hubei Key Laboratory of Genetic Regulation and Integrative Biology, School of Life Sciences, Central China Normal University, Wuhan, 430079 China

## Abstract

As the important source of natural fibers in the textile industry, cotton fiber quality and yield are often restricted to drought conditions because most of cotton plants in the world grow in the regions with water shortage. WRKY transcription factors regulate multiple plant physiological processes, including drought stress response. However, little is known of how the WRKY genes respond to drought stress in cotton. Our previous study revealed *GhWRKY33* is leaf-specific and induced by drought stress. In this study, our data showed GhWRKY33 protein localizes to the cell nucleus and is able to bind to “W-box” cis-acting elements of the target promoters. Under drought stress, *GhWRKY33* overexpressing transgenic Arabidopsis was withered much more quickly than wild type due to faster water loss. Moreover, *GhWRKY33* transgenic plants displayed more tolerance to abscisic acid (ABA), relative to wild type. Expression of some drought stress-related genes and ABA-responsive genes were changed in the *GhWRKY33* transgenic Arabidopsis with drought or ABA treatment. Collectively, our findings indicate that GhWRKY33 may act as a negative regulator to mediate plant response to drought stress and to participate in the ABA signaling pathway.

## Introduction

Plants often suffer from multifarious biotic and abiotic stresses with a sessile lifestyle. Drought is one of the most devastating natural factors which largely restrict growth and yield of crop plants all over the world. In order to survive, plants have evolved a series of tolerance mechanisms through increasing water uptake or reducing water loss to adapt and respond to these unfavorable environments. Under drought stress, guard cell signaling begins to function for reducing water loss. Meanwhile, the biosynthesis and accumulation of the important phytohormone abscisic acid (ABA) are increased straightly in plants cells. Expression levels of various stress-related genes are modulated by a number of transcription factors for regulating plant drought response^1–4^.

WRKY transcription factors play important roles to modulate diverse plant physiological processes by forming integral parts of signaling webs^5^. The WRKY family protein is defined by the most prominent domain which is characterized by a highly conserved WRKYGQK heptapeptide at its N-terminal and an atypical zinc finger-like motif at its C-terminal^6^. Generally, the WRKY family proteins are classified into three categories (I, II and III) due to the number and diversity of WRKY domains. There are two WRKY domains in Group I proteins and one in the others. Group II and Group III proteins are distinguished according to the different structure of the zinc fingers (C2H2 in Group II proteins and C2HC in Group III proteins)^6–8^. Furthermore, the Group II WRKY proteins can be divided into subgroups IIa, IIb, IIc, IId and IIe on the basis of the structure of other conserved primary amino acid sequence except the WRKY domains^8^. Since the first WRKY gene (*SPF1*) was cloned from sweet potato^9^, a large number of WRKY genes have been identified in more than 20 plant species up to now. Previous study reported 74 members in Arabidopsis WRKY family and more than 100 WRKY proteins in rice (*Oryza sativa*)^6^. Almost all WRKY transcription factors show specificity to bind preferentially to the W-box [TTGAC(C/T)] of promoters of their target genes, and thus regulate the expression of the downstream genes^10^.

WRKY transcription factors act as the vital regulator to function in manifold plant developmental and physiological processes. For example, Arabidopsis WRKY6 directly down-regulates *RAV1* expression to act as a positive regulator of ABA signaling during seed germination and early seedling development^11^. Arabidopsis TTG2 (a WRKY transcription factor) is involved in regulation of *GL2* transcription in epidermal cell differentiation^12^. AtWRKY54 and AtWRKY70 co-operate to negatively regulate leaf senescence^13^. WRKY transcription factors also play a vital role in complex signaling processes during plant biotic and abiotic stress responses^5,14–16^. For instance, heterologous expression of *OsWRKY23* gene could enhance pathogen defense^17^. Overexpression of *ZmWRKY58* enhances the drought and salt tolerance in transgenic rice^18^. Drought-responsive WRKY transcription factor genes *TaWRKY1* and *TaWRKY33* confer the transgenic Arabidopsis plants drought and/or heat resistance^19^. Moreover, *AtWRKY46* plays dual roles in regulating plant responses to osmotic stress and stomatal movement^20^, as well as coordinating with *WRKY70* and *WRKY53* in basal resistance against pathogen^21^.

ABA is a key component in response to various biotic and abiotic stresses. It can modulate large numbers of ABA-responsive genes and thus regulate many physiological processes^22,23^. In addition, WRKY transcription factors have been reported to be involved in drought stress through the ABA signaling pathway^24^. A recent study reported that *CmWRKY1* enhances the drought tolerance through regulating ABA-associated genes^25^. Moreover, *GhWRKY17* reduces the transgenic tobacco (*Nicotiana benthamiana*) tolerance to drought through modulating the ABA signaling pathway and reactive oxygen species (ROS) production^26^. The WRKY transcription factor, ABO3, functions in the ABA response and drought tolerance of Arabidopsis plants^27^.

Cotton (*Gossypium hirsutum*) is one of the most important economic crops in the world and the direct source of natural fibers for textile industry. However, the yield of cotton is strongly limited by drought stress. Water is essential for every phase of cotton growth and development, and water shortage (drought) would disturb leaf photosynthesis, plant water relation, nutrient relation, biological yield, seed and lint (fiber) yield of cotton. It was reported that drought stress leads to mean reduction of 42% in seed yield and 55% in biological yield of cotton^28,29^. Therefore, it is meaningful to understand the molecular mechanism how cotton plant responds to drought stress. In our previous study, we identified a group III WRKY gene, *GhWRKY33* in cotton, and revealed this gene is specifically expressed in leaves and induced by drought stress and ABA^30^. In this study, we demonstrated that GhWRKY33 protein is localized in the cell nucleus and could bind to the W-box elements. Overexpression of *GhWRKY33* enhances transgenic Arabidopsis plant drought sensitivity. Moreover, under drought stress, the transcription levels of some genes involved in drought stress were also altered in the *GhWRKY33* overexpressing transgenic Arabidopsis. Our results suggested that GhWRKY33 may act as a negative regulator to participate in plant drought response and the ABA signaling.

## Results

### GhWRKY33 functions as a transcription factor

**Figure 1:**
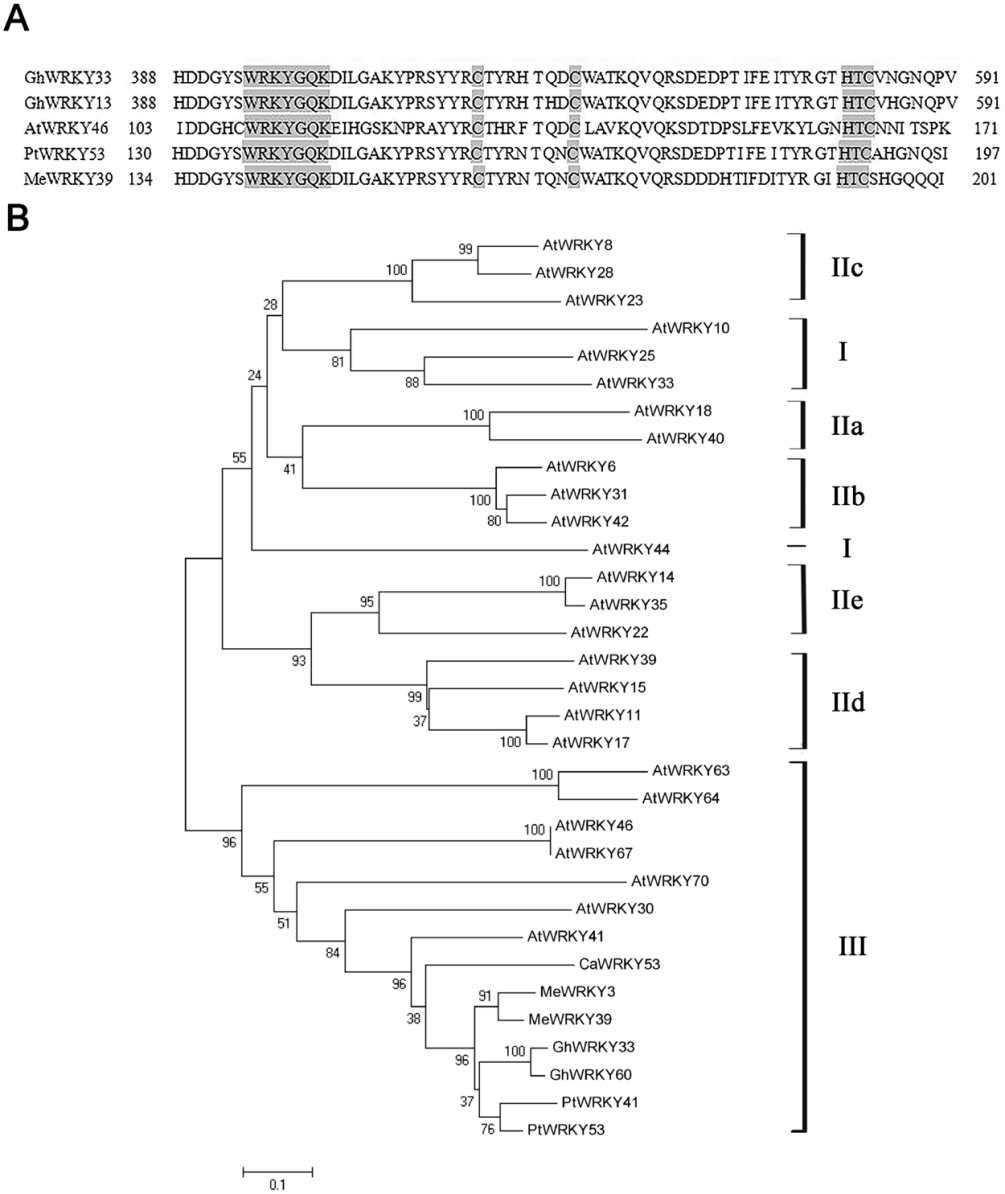
Sequence and phylogenetic analysis of GhWRKY33. (**A**) Sequence alignment of the amino acid sequence of GhWRKY33 with GhWRKY13 (KJ825862), AtWRKY46 (NP_182163), PtWRKY53 (EEE79528), and MeWRKY39 (KT827614). Conserved WRKY domain and zinc-finger motif are shown in grey. (**B**) Phylogenetic relationship of WRKY proteins from different plant species. All the 33 WRKYs protein sequences were subjected to Clustal W using the neighbor-joining method in MEGA 5 and can be divided into three groups (I, II, III), and Group II is further divided into five subgroups (IIa, IIb, IIc, IId, IIe). All the WRKY proteins used for the phylogenetic tree are: AtWRKY6 (NP_564792), AtWRKY8 (NP_199447), AtWRKY10 (NP_175956), AtWRKY11 (NP_567878), AtWRKY14 (NP_564359), AtWRKY15 (NP_179913), AtWRKY17 (NP_565574), AtWRKY18 (NP_001031766), AtWRKY22 (NP_192034), AtWRKY23 (NP_182248), AtWRKY25 (NP_180584), AtWRKY28 (NP_193551), AtWRKY30 (NP_568439), AtWRKY31 (NP_567644), AtWRKY33 (NP_181381), AtWRKY35 (NP_ 001324223), AtWRKY39 (NP_ 001030634), AtWRKY40 (NP_178199), AtWRKY41 (NP_192845), AtWRKY42 (NP_192354), AtWRKY44 (NP_001078015), AtWRKY46 (NP_182163), AtWRKY63 (NP_176833), AtWRKY64 (NP_176829), AtWRKY67 (NP_001117559), AtWRKY70 (NP_191199), CaWRKY53 (NP_001311621), GhWRKY33 (AJT43313), GhWRKY60 (AIE43835), PtWRKY53 (EEE79528), PtWRKY41 (XP_002297983), MeWRKY3 (AMO00371) and MeWRKY39 (KT827614). Gh, *Gossypium hirsutum*; At, *Arabidopsis thaliana*; Pt, *Populus trichocarpa*; Me, *Manihot esculenta*; Ca, *Capsicum annuum*.

In our previous study, *GhWRKY33* (GeneBank accession number: KJ825875) was identified in cotton^30^. The *GhWRKY33* gene contains a complete open reading frame (ORF) of 1086 bp that encodes a protein with 361 amino acids. Alignment analysis of GhWRKY33 protein sequence with its homologous sequences, including GhWRKY13 (KJ825862), AtWRKY46 (NP_182163), PtrWRKY53 (EEE79528) and MeWRKY39 (KT827614), showed that GhWRKY33 contains only one WRKY domain (WRKYGQK) and a C2HC zinc finger motif (Fig. 1A). Further phylogenetic analysis showed that GhWRKY33 displays the highest similarity to GhWRKY60. In addition, GhWRKY33 exhibited higher similarity to PtWRKY41 and PtWRKY53 from *Populus tremula*, besides AtWRKY41 in *Arabidopsis* (Fig. 1B). These results suggested that GhWRKY33 is a member of group III WRKY transcription factors.

**Figure 2:**
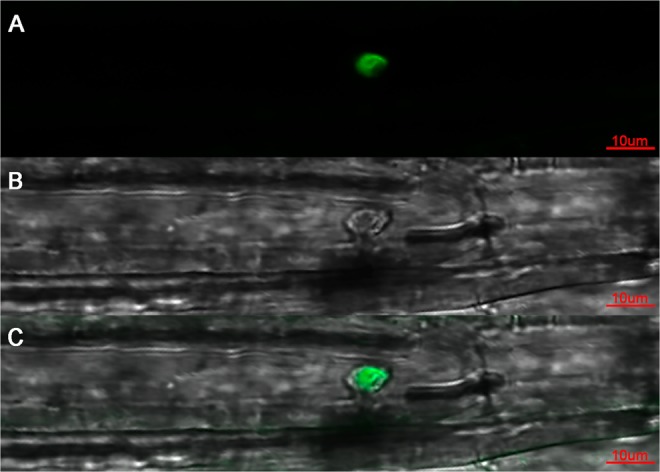
Subcellular localization of GhWRKY33 protein. Confocal images were taken of the hypocotyl cells of 35S:GhWRKY33-GFP transgenic Arabidopsis. (**A**) Confocal image of the hypocotyl cell under the GFP channel. (**B**) The transmitted light image of the same cell in image A. (**C**) The merged images of confocal image and the bright-field image. Bar = 10 μm.

To analyze the intracellular localization of GhWRKY33, we introduced the 35S:GhWRKY33:eGFP (enhanced green fluorescent protein) construct into Arabidopsis to generate the transgenic plants (see Methods). Confocal microscopy was used to detect the GFP fluorescence in the hypocotyls of T2 generation transgenic seedlings under GFP channel and transmitted light field. The results showed that the GFP fluorescence was seen mainly in the nuclei of cells (Fig. 2), indicating GhWRKY33 is the nuclear-localized protein.

**Figure 3:**
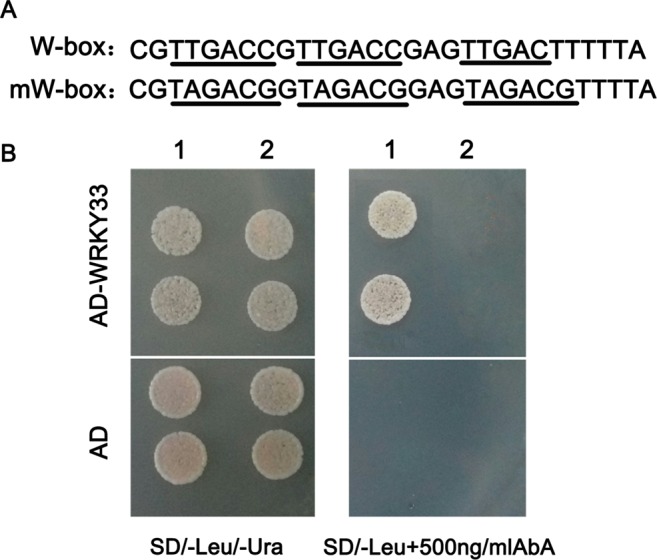
Characterization of GhWRKY33 as a transcriptional factor. (**A**) The sequence of the triple tandem repeats of the W-box and mW-box binding elements. (**B**) Yeast one-hybrid assay using the triple tandem repeats of the W-box and mW-box as bait. Yeast cells carrying pGAD-GhWRKY33 or pGAD7 were grown on SD/-Leu/-Ura or SD/-Leu containing 500 ng/ml AbA. 1: pAbAi-W-box; 2: pAbAi-mW-box.

Previous studies reported that the WRKY transcription factors modulate target genes expression through binding to the W-box [TTGAC(C/T)] in the promoters of downstream target genes^31^. Hence, we determined whether GhWRKY33 protein has the ability to bind to the cis-element by yeast one-hybrid assay. As shown in Fig. 3A, three tandem repeats of the W-box (TTGACC) or mW-box (TAGACG) were inserted into the pAbAi vector, which contains the Aureobasidin A resistance (AbA^r^) reporter gene (AUR-1C), and integrated into the genome of the yeast strain Y1HGold. Meanwhile *GhWRKY33* gene was cloned into pGADT7 to form a yeast effector vector, pGADT7-WRKY33. Then pGADT7-WRKY33 and pGADT7 constructs were transformed into the Y1HGold yeast strain carrying the pAbAi-W-box or pAbAi-mW-box plasmids. To verify the success of transformation, the transformed yeast cells grew on leucine (Leu) and uracil (Ura)-deficient synthetic dextrose (SD) medium (SD/-Leu/-Ura), respectively (Fig. 3B). Furthermore, only the yeast clones with pAbAi-W-box and pGAD-GhWRKY33 grew on SD/-Leu containing 500 ng/ml AbA. AbA^r^ basal expression assays displayed that the concentration of AbA could suppress the basal expression of the pAbAi-W-box/ pAbAi-mW-box reporter strain in the absence of prey (Fig. 3B). Above results indicated that GhWRKY33 could bind to the W-box element and might function as a transcription factor to modulate expression of its target genes.

### Overexpression of *GhWRKY33* enhances transgenic plant sensitivity to drought stress

**Figure 4:**
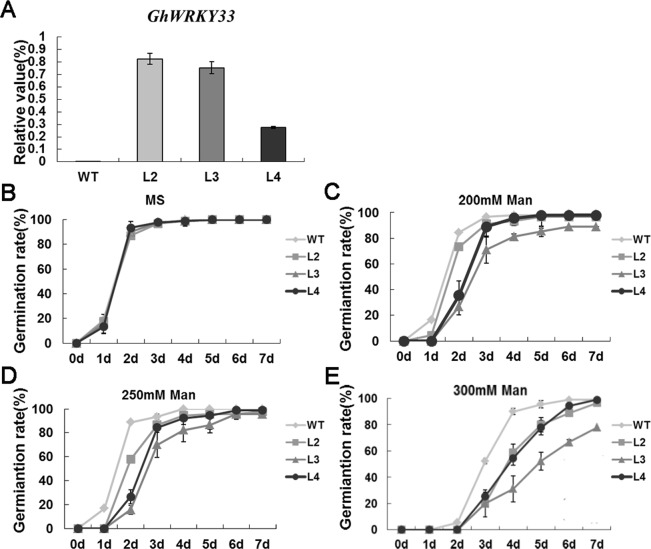
Assay of seed germination rate of *GhWRKY33* overexpression transgenic Arabidopsis with mannitol treatment. (**A**) Quantitative RT-PCR analysis of *GhWRKY33* expression in the transgenic lines and wild type. (**B–E)** Statistical analysis of seed germination rate. Seeds of the transgenic lines and wild type were germinated on MS medium (**B**), MS medium with 200 mM mannitol (**C**), MS medium with 250 mM mannitol (**D**), and MS medium with 300 mM mannitol (**E**). Each curve represents an average of three replicates. WT, wild type; L2, L3 and L4, three *GhWRKY33* overexpression transgenic lines.

**Figure 5:**
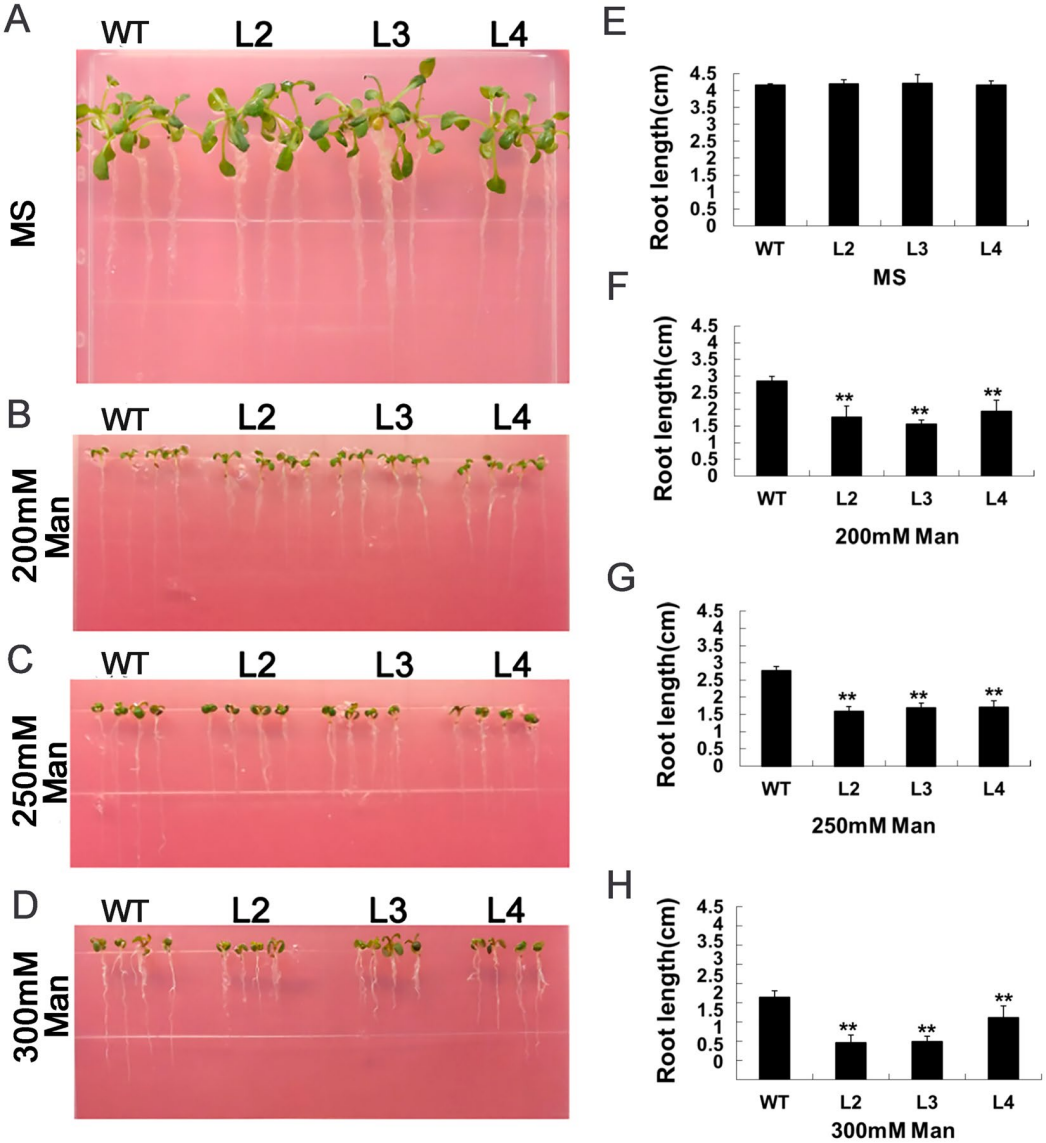
Assay of root length of *GhWRKY33* overexpression transgenic Arabidopsis with mannitol treatment. (**A–D)** Seedlings of wild type and *GhWRKY33* transgenic lines grew on MS medium (**A**), MS medium with 200 mM mannitol (**B**), MS medium with 250 mM mannitol (**C**), and MS medium with 300 mM mannitol (**D**). **(E–H)** Statistical analysis of root length of wild type and *GhWRKY33* overexpression transgenic seedlings grown on MS medium with or without mannitol treatment. Seeds germinated on MS medium for 3 days and then the seedlings were transferred onto MS medium containing different concentration of mannitol for 7 days. Mean values and standard errors (bars) are shown from three independent experiments. Two asterisks represent there was a very significant difference (P < 0.01) in root length between the transgenic lines and wild type. MS, seedlings grew on MS medium without Man as control; 200 mM Man, 250 mM Man, 300 mM Man, seedlings grew on MS medium with 200, 250, and 300 mM mannitol, respectively. WT, wild type; L2, L3 and L4, three *GhWRKY33* overexpression transgenic lines.

Our previous study revealed that *GhWRKY33* is specifically expressed in cotton leaves. When cotton was treated with drought and ABA, the expression level of *GhWRKY33* was up-regulated in cotton^30^. Thus we speculated *GhWRKY33* might play a role when cotton responds to drought and ABA signaling. Therefore, *GhWRKY33* was introduced to ectopically express in *Arabidopsis thaliana* as this model plant has been extensively used for understanding plant stress-response. We used T3 generation homozygous transgenic plants (L2, L3 and L4 lines with different transcription levels of the *GhWRKY33* gene) as the further experimental materials (Fig. 4A). First of all, seeds of wild type and overexpression transgenic lines were put on Murashige and Skoog (MS) medium with 0, 200, 250 and 300 mM mannitol, respectively, for germination. We found the seed germination rate of *GhWRKY33* overexpressing transgenic Arabidopsis was similar to that of wild type without mannitol treatment. When treated with mannitol, on the contrary, the seed germination rate of the transgenic Arabidopsis overexpressing *GhWRKY33* was decreased, compared with wild type (Fig. 4B–E). After 3 days in the presence of 300 mM mannitol, about 50% of wild type seeds were germinated, whereas only nearly 20% of transgenic seeds germinated (Fig. 4E). Thereafter, the seedlings were transferred to MS medium containing mannitol (0, 200, 250 and 300 mM) in the vertical position for 7 days to calculate the root length. The results showed that root growth of the transgenic Arabidopsis overexpressing *GhWRKY33* was significantly inhibited, compared with wild type controls when these seedlings were treated with different concentrations of mannitol (Fig. 5). These data indicated that *GhWRKY33* transgenic Arabidopsis plants displayed drought sensitivity during seed germination and early seedling growth.

**Figure 6:**
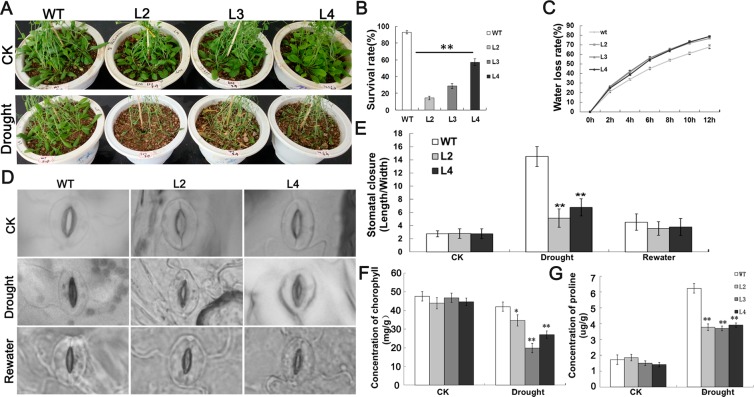
Assay of *GhWRKY33* overexpression transgenic Arabidopsis tolerant to drought stress. (**A**) phenotypic analysis of 3-week-old wild type and *GhWRKY33* transgenic Arabidopsis grown in soil under normal conditions (top) or drought stress by withholding water for 10 days (bottom). (**B**) Survival rates of the plants shown in (A) after re-watering. (**C**) Water loss rate of the detached leaves of *GhWRKY33* overexpression transgenic lines and wild type. (**D**) Stomatal closure of transgenic lines and wild type with or without drought stress were observed. (**E**) Measurement and statistical analysis of stomatal closure (stomatal length:width ratio) under drought treatment (n > 40 for each sample). (**F**) Assay of chlorophyll content in leaves of *GhWRKY33* overexpression transgenic lines and wild type under normal conditions and under drought stress. (**G**) Assay of proline accumulation in leaves of *GhWRKY33* overexpression transgenic lines and wild type under normal conditions and under drought treatment. Mean values and standard errors (bars) are shown from three independent experiments. One and two asterisks represent there was a significant difference (P < 0.05) and a very significant difference (P < 0.01) in Duncan’s multiple range test, respectively. CK, seedlings grew in soil under normal conditions as controls; Drought, seedlings grew in soil kept from water (under drought stress); WT, wild type; L2, L3 and L4, three *GhWRKY33* overexpression transgenic lines.

Additionally, we further studied how *GhWRKY33* functions in drought response during vegetative growth of the transgenic plants. Three-week-old, soil-grown *GhWRKY33* transgenic Arabidopsis seedlings and wild type controls were kept away from water for 10 days. As shown in Fig. 6A, *GhWRKY33* overexpression transgenic plants appeared to be yellowing and more withered than wild type controls which were still green. After re-watered, less than 20% of transgenic plants were survived, but wild type had more than 80% survival rate (Fig. 6B). Meanwhile, leaves from *GhWRKY33* transgenic lines and wild type were detached from plants and weighed an hourly within 12 hours. We assumed weight loss is due to water loss from the leaves. In this experiment, the rate of water loss from leaves of *GhWRKY33* overexpressing Arabidopsis was obviously higher relative to the wild type controls (Fig. 6C).

When plants confront drought stress, the stomata serves as a vital gateway to limit water loss^32^. Therefore, stomatal conditions of *GhWRKY33* transgenic lines and wild type were observed by microscopy with or without drought treatment. Under natural growth conditions, the *GhWRKY33* transgenic Arabidopsis showed no significant difference in the ratio of stomatal length to width, compared with wild type controls. When exposed to drought, in contrast, the stomata of *GhWRKY33* transgenic lines opened wider than those of wild type (Fig. 6D,E), indicating that *GhWRKY33* overexpressing transgenic lines present higher water loss rate, mainly owing to the impaired stomatal closure.

It has been suggested that environment stressors (including drought stress) may influence chlorophyll and proline contents in plants. Thus, we further determined the chlorophyll and proline contents in *GhWRKY33* overexpressing transgenic Arabidopsis leaves grown with or without drought stress, using the wild type as controls. The experimental results revealed that total chlorophyll content and proline accumulation in *GhWRKY33* overexpressing transgenic leaves were as same high as wild type without drought stress. In contrast, the contents of chlorophyll in *GhWRKY33* overexpressing transgenic leaves were nearly 15% lower compared with the wild type, and proline accumulation in the transgenic plants reduced approximately 40% relative to wild type controls with drought treatments (Fig. 6F,G). The above data implied that overexpression of *GhWRKY33* enhances transgenic *Arabidopsis* sensitivity to drought stress.

### Overexpression of *GhWRKY33* reduces the transgenic plant sensitivity to ABA

**Figure 7:**
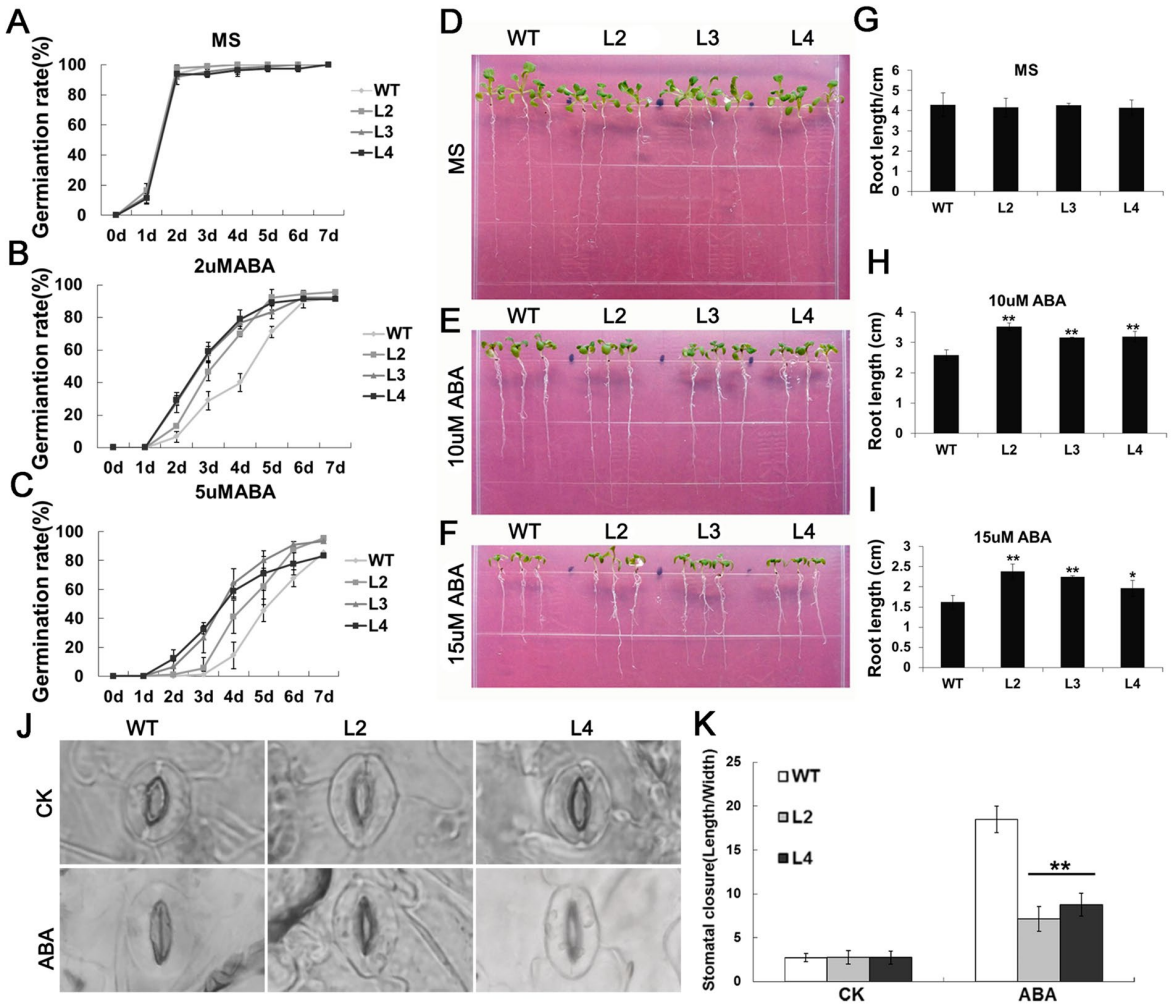
Assay of GhWRKY33 overexpression transgenic Arabidopsis tolerant to ABA. (**A**–**C**) Seed germination rates of *GhWRKY33* transgenic lines and wild type with different ABA concentrations (0, 2 and 5 μM). Germination was recorded daily. (**D**–**F**) Seedlings of transgenic lines and wild type with 0, 10 and 15 μM ABA treatment. (**G**–**I**) Statistical analysis of the root length of seedlings with 0, 10 and 15 μM ABA treatment. (**J**) Micrographs of stomatal changes of the transgenic lines and wild type with or without 20 μM ABA treatment. (**K**) Measurement of statistical analysis of stomatal closure (stomatal length:width ratio) of the transgenic lines and wild type with or without ABA treatment (n > 40 for each sample). Mean values and standard errors (bars) are shown from three independent experiments. One and two asterisks represent there was a significant difference (P < 0.05) and a very significant difference (P < 0.01) in Duncan’s multiple range test, respectively. CK, seedlings grew under normal conditions as controls; ABA, seedlings grew with 20 μM ABA treatment; WT, wild type; L2, L3 and L4, three *GhWRKY33* overexpression transgenic lines.

ABA functions as a crucial phytohormone in complex signaling networks and is involved in regulating plant development and stress response^33^. When plants confront with drought stress, ABA is synthesized to induce stomatal closure in order to prevent water loss by transpiration^34^. To find whether *GhWRKY33* plays a role in ABA signaling, both *GhWRKY33* transgenic Arabidopsis and wild type seeds germinated with 0, 2 and 5 μM ABA treatments, respectively. The results showed that seed germination rate of the *GhWRKY33* overexpressing transgenic Arabidopsis was increased, compared with wild type, under ABA treatment (Fig. 7A–C). When exogenous ABA is present, additionally, roots of the *GhWRKY33* overexpressing transgenic Arabidopsis grew obviously better, relative to the controls. when exogenous ABA does not exist, on the other hand, the root growth of the *GhWRKY33* overexpressing transgenic Arabidopsis and wild type was almost identical (Fig. 7D–I). These data indicated *GhWRKY33* overexpressing transgenic Arabidopsis showed a reduced sensitivity to ABA during seed germination and early seedling development.

To further study the connection between GhWRKY33 transcription factor and ABA-induced stomatal closure, the change in stomatal aperture was analyzed in the transgenic plants relative to wild type. In the presence of 20 μM ABA, the stomata in wild type leaves were almost completely closed, while the stomata of the *GhWRKY33* transgenic lines were still open. The *GhWRKY33* overexpressing transgenic Arabidopsis plants displayed the decreased ratio of stomatal length to width, compared with the wild type (Fig. 7J,K). The above results suggested that ABA-induced stomatal closure is impaired in *GhWRKY33* overexpression transgenic Arabidopsis plants, implying GhWRKY33 might play a negative regulator of ABA signaling.

### GhWRKY33 negatively regulates drought-related and ABA-responsive genes in transgenic Arabidopsis

**Figure 8:**
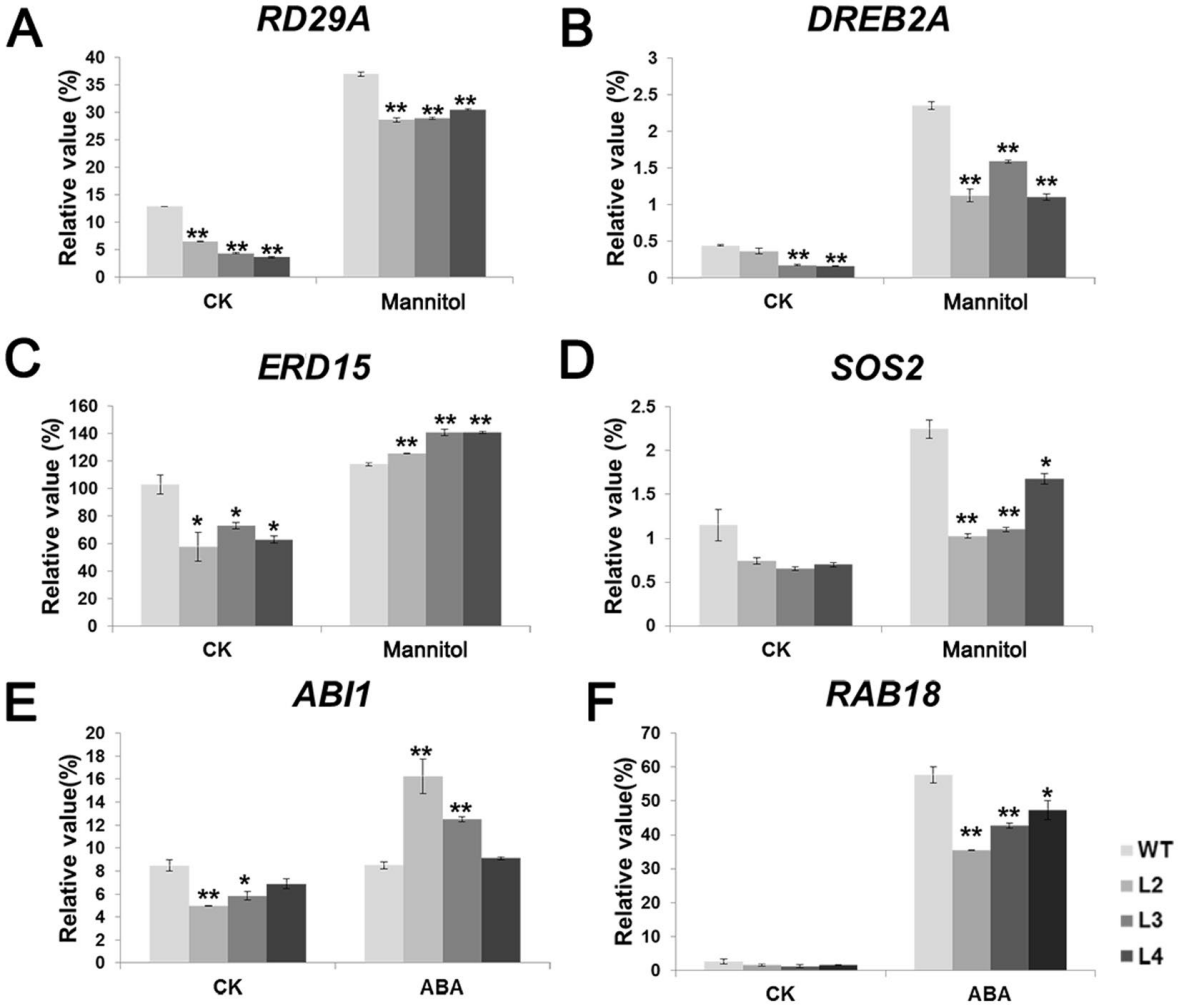
Quantitative RT-PCR analysis of expression of the drought stress-related and ABA-responsive genes in *GhWRKY33* transgenic Arabidopsis. Total RNA was isolated from 10-day-old seedlings grown without (CK) or with 250 mM mannitol treatment for 72 h or with 100 μM ABA treatment for 6 h. Transcript levels of *RD29A, DREB2A, ERD15, SOS2, RAB18* and *ABI1* in the transgenic lines and wild type were determined by quantitative RT-PCR using *AtACTIN2* as a quantification control. Mean values and standard errors (bars) were shown from three independent experiments. One and two asterisks represent there was a significant difference (P < 0.05) and a very significant difference (P < 0.01) in gene expression level between the transgenic lines and wild type, respectively. CK, seedlings grew under normal conditions; Mannitol, seedlings with mannitol treatment; ABA, seedlings with ABA treatment; WT, wild type; L2, L3, L4, three *GhWRKY33* transgenic lines.

The above work indicated that GhWRKY33 might be a negative regulator involved in plant response to drought stress and ABA signaling pathway. Therefore, we analyzed expression of several drought-related genes (such as *RD29A*, *DREB2A, ERD15, SOS2*) in *GhWRKY33* overexpression transgenic plants and wild type with or without drought stress. As shown in Fig. 8A–D, under normal conditions, expression of all these genes was reduced in the *GhWRKY33* overexpressing transgenic Arabidopsis compared with the wild type controls. After drought treatment, the expression levels of these tested genes were remarkably increased in the *GhWRKY33* overexpressing Arabidopsis and wild type controls. Compared with the wild type, however, the expression level of *ERD15* was higher, whereas the expression levels of *RD29A*, *DREB2A* and *SOS2* were still lower in the *GhWRKY33* overexpressing Arabidopsis. Moreover, we also analyzed the expression of two ABA-responsive genes (*ABI1* and *RAB18*) in transgenic plants and wild type with or without ABA treatment. The results showed that under normal conditions, the expression levels of *ABI1* and *RAB18* in the *GhWRKY33* overexpressing transgenic Arabidopsis were decreased, relative to the wild type controls. When exogenous ABA exists, on the contrary, the expression level of *ABI1* in the transgenic plants was remarkably enhanced, but *RAB18* expression in the *GhWRKY33* overexpressing Arabidopsis was declined compared with the wild type controls (Fig. 8E,F).

**Figure 9:**
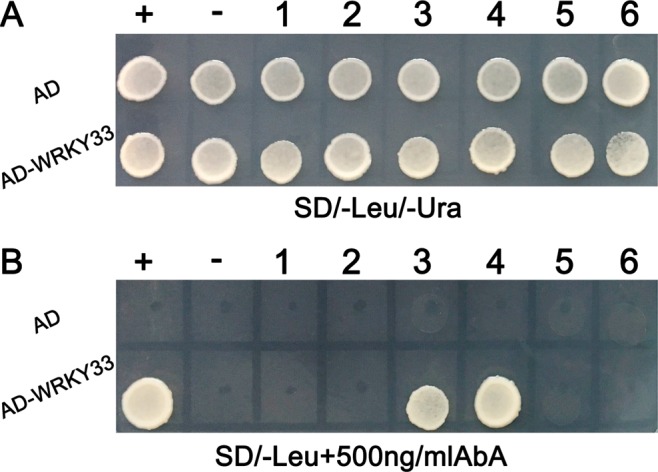
Yeast one-hybrid assay of GhWRKY33 binding to promoters of the drought stress-related and ABA-responsive genes. Yeast cells carrying pGAD-GhWRKY33 or pGAD7 were grown on SD/-Leu/-Ura or SD/-Leu containing 500 ng/ml AbA. + , pAbAi-W-box; −, pAbAi-mW-box; 1, pAbAi-RD29Apro; 2, pAbAi-DREB2Apro; 3, pAbAi-ERD15pro; 4, pAbAi-SOS2pro; 5, pAbAi-ABI1pro; 6: pAbAi-RAB18pro.

To further investigate how GhWRKY33 regulates these downstream genes, we analyzed the promoter regions of these marker genes with the PlantCARE databases. Two, three, two, ten, three and one putative W-box cis-elements were found in the up-stream sequences of *RD29A*, *DREB2A*, *ERD15*, *SOS2*, *ABI1* and *RAB18*, respectively. Then a yeast one-hybrid assay was performed to determine whether the GhWRKY33 protein could bind to the promoters of these downstream genes. As shown in Fig. 9, GhWRKY33 could bind to the promoters of *ERD15* and *SOS2*. Based on the above data, we speculated that GhWRKY33 may directly or indirectly regulate expression of drought-related and ABA-responsive genes by binding to the W-box in the promoter regions of them, so that in response to drought stress and ABA signaling.

## Discussion

Owing to the characteristics of the sessile lifestyle, plants have to develop a complex regulatory circuitry in response to biotic and abiotic stresses. The regulatory circuitry is comprised of transcriptional activators and repressors to modulate the expression of defense genes. It has been reported that numerous transcription factor families participate in regulating the defense transcriptome^35^. In recent years, WRKY transcription factors are reported as pivotal regulators of the defense transcriptome in drought stress. For example, *AtWRKY53* takes part in drought response through mediating stomatal movement^36^. Overexpression of *CmWRKY10* in chrysanthemum enhances plant drought resistance through ABA-signaling pathway^37^. GhWRKY59, which is phosphorylated and activated by GhMPK6, is involved in regulating cotton drought responses^38^. Furthermore, overexpression of *GhWRKY68* in tobacco (*Nicotiana benthamiana*) reduces transgenic plant resistance to drought response by modulating ABA signaling^39^. In our previous study, 26 WRKY genes were identified in cotton. Among these WRKY genes, *GhWRKY33* showed specific expression in leaves and could be induced by drought stress and ABA treatment^30^. These data provided us a feasible clue that *GhWRKY33* may participate in drought responses and ABA signaling pathway. In this study, we revealed that GhWRKY33 is a member of group III WRKY transcription factors. *GhWRKY33* overexpressing transgenic plants were more sensitive to drought stress than wild type. It has been all known that stomatal closure represents an early response to drought stress to maintain the internal osmotic environment^40^. Under drought stress, however, *GhWRKY33* transgenic plants still maintained the wider stomatal aperture than wild type thus causing the higher rate of water loss. Both chlorophyll content and proline accumulation were remarkably reduced in leaves of the transgenic plants. These data provided us a revelation that GhWRKY33 may participate in drought responses by mediating the stomatal movement.

It has been reported that reduced drought resistance accompanies hyposensitive to ABA during seed germination and early seedling growth^41^. ABA is a critical signaling molecule with various biological functions, such as regulating guard cell volume, maintaining seed dormancy, preventing germination and inhibiting seedling growth^42,43^. Previous study revealed that AtYY1 is a negative regulator of the ABA response network and loss of *AtYY1* enhanced ABA-induced stomatal closing and drought resistance^44^. *AtYak1* knockout mutant plants were hyposensitive to ABA inhibition of seed germination, seedling growth and stomatal movement, suggesting AtYak1 as a positive regulator plays an important role in ABA-mediated drought response^41^. To clarify whether *GhWRKY33* responds to drought in ABA-mediated signaling pathway, similarly, the *GhWRKY33* transgenic Arabidopsis and wild type plants were exposed to exogenous ABA to compare difference of their growth status. Our findings indicated that *GhWRKY33* transgenic plants were less sensitive to ABA than wild type during seed germination and seedling growth. Moreover, the transgenic plants also reduced the sensitivity of ABA-induced stomatal closure. These results indicated that *GhWRKY33* reduced drought resistance by acting as a negative regulator in ABA signaling pathway.

Transcription factors regulate the expression of the stress-related target genes to participate in various plant stresses^5^. For instance, ectopic expression of *GaMYB85* enhanced transcript levels of stress-related marker genes (such as *RD22, AD11, RD29A* and *ABI5*) to improve Arabidopsis tolerance to drought stress^45^. *GsWRKY20* plays an important role in enhancing plant drought tolerance and regulating ABA signaling through promoting the expression of *ABI1, ABI2, ABI4, ABI5* and *ABF4*^46^. Similarly, we analyzed expression of several drought stress-related and ABA-responsive genes in *GhWRKY33* overexpressing transgenic Arabidopsis and wild type controls. Previous studies revealed that Arabidopsis *RD29A* is a drought- or ABA-induced gene that could rapidly respond to drought stress in ABA-independent manner^47–49^. *DREB2A* that is induced by dehydration regulates expression of many dehydration-inducible genes^49^. *EARLY RESPONSIVE TO DEHYDRATION 15* (*ERD15*) was reported as the key negative regulators of ABA responses and a rapidly drought-responsive gene in Arabidopsis. Overexpression of *ERD15* decreases tolerance to drought and reduces sensitivity to ABA^50^. *SOS2* has been reported with an effect on improving drought stress resistance^51^. In this study, the expression levels of *RD29A*, *DREB2A* and *SOS2* in the *GhWRKY33* overexpressing transgenic Arabidopsis plants were remarkably declined compared with the wild type controls with or without drought treatment. But *ERD15* in *GhWRKY33* transgenic lines exhibited a higher expression level than that in wild type with drought treatment. In addition, two ABA-responsive genes *RAB18* and *ABI1* were also analyzed. Previously reported that *RAB18* is a typical ABA-dependent stress-responsive gene and could be induced by ABA-associated drought stress^52^. The type 2C protein phosphatase ABI1 (ABA INSENSITIVE 1) acts as a key negative regulator of ABA signaling and plays a negative role in the ABA-induced stomatal closure^53–56^. Our results showed the expression levels of *RAB18* and *ABI1* were induced in both wild type and *GhWRKY33* transgenic plants with exogenous ABA treatment. Furthermore, the expression level of *RAB18* was lower in *GhWRKY33* transgenic lines than that in wild type, whereas the relatively higher transcript level of *ABI1* was found in the transgenic plants relative to wild type under exogenous ABA treatment, suggesting that GhWRKY33 may act as a negative regulator of ABA signaling. WRKY transcription factors were reported to regulate the expression of target genes by binding to W-box in their promoter regions. Hence, we analyzed the promoter sequences of *RD29A, DREB2A, ERD15, SOS2, ABI1* and *RAB18* with the PlantCARE analysis databases, and found the W-box cis-elements existed in the up-stream sequences of *RD29A*, *DREB2A, ERD15*, *SOS2*, *ABI1* and *RAB18*. Furthermore, yeast one-hybrid assay confirmed GhWRKY33 could bind to the promoters of *ERD15* and *SOS2*. So we deduced that GhWRKY33 may respond to drought stress and ABA signaling by directly or indirectly regulating the drought stress-related and ABA-responsive genes. Collectively, our data revealed that GhWRKY33 may negatively regulate plant response to drought stress via modulating ABA signaling and stomatal closure.

## Materials and Methods

### Plant materials and growth conditions

Seeds of *Arabidopsis thaliana* (*Columbia* ecotype) were surface-sterilized with 10% NaClO for 5 min, followed by washing three times with sterile water. The sterilized *Arabidopsis* seeds were plated on Murashige and Skoog (MS) medium. After placed at 4 °C for 72 hours in darkness, these seeds were transferred into the growth incubator for germination and development (22 °C, 16 hours light/8 hours dark). Seven days later, the seedlings were transplanted into soil and grew in the growth chamber (22 °C, 16 hours light/8 hours dark). Tissues were harvested from these seedlings for further study.

### GhWRKY33 sequence analysis

The *GhWRKY33* cDNA (KJ825875) was identified from cotton cDNA libraries^30^. Homologous sequences of *GhWRKY33* were retrieved from the NCBI databases and sequence alignment was performed with ClustalX. MEGA5 program was used to generate the phylogenetic tree of GhWRKY33 and bootstrap analysis to assess the statistical reliability. The PlantCARE was used to analyze the promoter sequences of drought-related genes.

### Subcellular localization

The open reading frame (ORF) of *GhWRKY33* was inserted into the binary vector *pBI121-eGFP* with an eGFP gene to generate *pBI121-GhWRKY33-eGFP* vector. The construct was introduced into *Arabidopsis* by *Agrobacterium*-mediated floral-dip method. The transformed seeds grown on the MS medium for 5 days were used for observing GFP fluorescence in hypocotyl cells under a SP5 Meta confocal laser microscope (Leica, Germany)^57^. The gene-specific primers are listed in Table S1.

### RNA extraction and quantitative RT-PCR analysis

Total RNA was extracted from Arabidopsis seedlings with Trizol reagent (Invitrogen) with Trizol reagent (Invitrogen) according to the manufacture protocol, and treated with DNase I (Takara) at 37 °C for 1 hour to eliminate genomic DNA contamination. The cDNA was reversely synthesized from the treated RNA, and used as real-time PCR templates with gene-specific primers. The PCR amplification was performed in the detection system (Opticon2; MJ Research, New Haven, Connecticut, USA) according to the method described earlier^58^, using *AtACTIN2* gene as an internal reference. Each qRT-PCR analysis was repeated three times. Mean values and standard variations were estimated from the data of three biological experiments. All primers used in the experiments are listed in Table S1.

### Arabidopsis transformation and phenotypic analysis of the transgenic *Arabidopsis*

The coding sequence of *GhWRKY33* gene was cloned into pMD vector at *Bam*H I and *Sac* I sites to generate 35S:GhWRKY33 construct. Then, the construct was introduced into Arabidopsis through *Agrobacterium*-mediated DNA transformation. Seeds of *GhWRKY33* homozygous lines (T3 generation) were selected by kanamycin resistance and were used for further study. All primers used in the experiments are listed in Table S1.

For mannitol and ABA treatments, *GhWRKY33* overexpressing transgenic Arabiopsis and wild type seeds germinated on MS medium containing 0, 200, 250 and 300 mM mannitol or 0, 2 and 5 μM ABA, respectively^26,45^. Seed germination rate was evaluated daily (n > 100 seeds for each line). The root elongation assay was performed by the method as described previously^59^. Briefly, three-day-old Arabidopsis seedlings grew on MS medium containing 0, 200, 250 and 300 mM mannitol or 0, 10 and 15 μM ABA in a totally upright position for one week. Then, root length was measured and statistically analyzed (n > 30 seedlings for each line). All experiments were performed with three technical replications.

For drought treatment, one-week-old wild type and *GhWRKY33* overexpressing transgenic Arabidopsis seedlings grew in soil under the same long-day conditions with normal watering for 3 weeks. Subsequently, water was withheld for approximately 10 days and photographs were taken after plants were again watered. To assay water loss rate, twenty fully expanded leaves were detached from three-week-old plants of the wild type and *GhWRKY33* overexpressing transgenic Arabidopsis, and then weighed an hourly within 12 hours. The water loss rate was calculated relative to the initial fresh weights.

To measure proline content, leaves of Arabidopsis seedlings treated with or without 250 mM mannitol were reacted with a mixture of 3% sulphosalicylic acid, glacial acetic acid and 2.5% ninhydrin in boiling water for 1 hour, respectively. And then proline was extracted with toluol and measured absorbance at 520 nm with a spectrophotometer^60^.

To measure chlorophyll content, leaves of Arabidopsis seedlings grown under normal or drought conditions were extracted with 80% acetone, and then the absorbance of the extract was measured with a spectrophotometer at 645, 652 and 663 nm to determine the chlorophyll content^61^.

### Stomatal movement assay

To observe and measure stomatal aperture, rosette leaves of Arabidopsis plants were floated in a solution containing 50 μM CaCl_2_, 10 mM KCl, 10 mM MES-Tris (pH 6.15), and exposed to light for 2 hours. Then ABA was added to the solution up to 20 μM for 2 hours, and stomatal apertures were measured as described previously^62^. Meanwhile, stomatal changes were observed by microscopy after drought treatment, and the ratio of stomatal length to width was recorded. Each sample was replicated at least three times.

### Yeast one-hybrid assay

For protein-DNA binding assay, we use the Matchmaker Gold Yeast One-Hybrid Library Screening System (Clontech). Oligonucleotide sequences were synthesized containing triple tandem copies of the W-box (TTGACC). After annealed, these oligonucleotide sequences were inserted into the pAbAi vector which was introduced into the yeast strain Y1HGold, forming a W-box-specific reporter strain used as bait. Moreover, the promoter fragments of *RD29A*, *DREB2A*, *ERD15*, *SOS2*, *ABI1* and *RAB18* were cloned into pAbAi vector which was also transformed into Y1H Gold strain to generate a bait-specific reporter strain. The coding sequence of *GhWRKY33* was fused to the one-hybrid vector pGADT7 with the GAL4 activation domain to generate the pGAD-GhWRKY33 yeast expression vector. Subsequently, pGADT7 and pGAD-GhWRKY33 were transformed into the bait-specific reporter strain. These yeast cells were plated on SD/-Leu/-Ura medium containing a certain concentration of AbA, which could completely suppress the basal expression of the reporter strain in the absence of prey, for observing yeast growth condition. Mutant W-box (mW-box) (TAGACG) was used as a negative control^26^. All primers used in the experiments are listed in Table S1.

## Supplementary information

Supplementary information

## References

[1] Ingram J, Bartels D (1996). The molecular basis of dehydration tolerance in plants. Annu Rev Plant Physiol Plant Mol Biol.

[2] Zhu JK (2002). Salt and drought stress signal transduction in plants. Annu Rev Plant Biol.

[3] Xiong L, Schumaker KS, Zhu JK (2002). Cell signaling during cold, drought, and salt stress. Plant Cell.

[4] Tuteja N (2007). Abscisic acid and abiotic stress signaling. Plant Signal Behav.

[5] Eulgem T, Somssich IE (2007). Networks of WRKY transcription factors in defense signaling. Curr Opin Plant Biol.

[6] Eulgem T, Rushton PJ, Robatzek S, Somssich IE (2000). The WRKY superfamily of plant transcription factors. Trends Plant Sci.

[7] Ülker B, Somssich IE (2004). WRKY transcription factors: from DNA binding towards biological function. Curr Opin Plant Biol.

[8] Rushton PJ (2010). WRKY transcription factors. Trends Plant Sci.

[9] Ishiguro S, Nakamura K (1994). Characterization of a cdna-encoding a novel dna-binding protein, SPF1, that recognizes SP8 sequences in the 5′ upstream regions of genes-coding for sporamin and beta-amylase from sweet-potato. Mol Gen Genet.

[10] Ciolkowski I, Wanke D, Birkenbihl RP, Somssich IE (2008). Studies on DNA-binding selectivity of WRKY transcription factors lend structural clues into WRKY domain function. Plant Mol Biol.

[11] Huang Y (2016). Arabidopsis WRKY6 Transcription Factor Acts as a Positive Regulator of Abscisic Acid Signaling during Seed Germination and Early Seedling Development. PLoS Genet.

[12] Ishida T (2007). Arabidopsis TRANSPARENT TESTA GLABRA2 is directly regulated by R2R3 MYB transcription factors and is involved in regulation of GLABRA2 transcription in epidermal differentiation. Plant Cell.

[13] Besseau S, Li J, Palva ET (2012). WRKY54 and WRKY70 co-operate as negative regulators of leaf senescence in *Arabidopsis thaliana*. J Exp Bot.

[14] Ryu HS (2006). A comprehensive expression analysis of the WRKY gene superfamily in rice plants during defense response. Plant Cell Rep.

[15] Pandey SP, Somssich IE (2009). The role of WRKY transcription factors in plant immunity. Plant Physiol.

[16] Li H (2013). ZmWRKY33, a WRKY maize transcription factor conferring enhanced salt stress tolerances in Arabidopsis. Plant Growth Regul.

[17] Jing SJ (2009). Heterologous expression of OsWRKY23 gene enhances pathogen defense and dark-induced leaf senescence in Arabidopsis. Plant Growth Regul.

[18] Cai RH (2014). Overexpression of a maize WRKY58 gene enhances drought and salt tolerance in transgenic rice. Plant Cell Tiss Organ Cult.

[19] He GH (2016). Drought-responsive WRKY transcription factor genes TaWRKY1 and TaWRKY33 from wheat confer drought and/or heat resistance in Arabidopsis. BMC Plant Biol.

[20] Ding ZJ (2014). Zheng, S. J. Transcription factor WRKY46 regulates osmotic stress responses and stomatal movement independently in Arabidopsis. Plant J.

[21] Hu YR, Dong QY, Yu DQ (2012). Arabidopsis WRKY46 coordinates with WRKY70 and WRKY53 in basal resistance against pathogen Pseudomonas syringae. Plant Sci.

[22] Chen H (2010). Roles of Arabidopsis WRKY18, WRKY40 and WRKY60 transcription factors in plant responses to abscisic acid and abiotic stress. BMC Plant Biol.

[23] Raghavendra AS (2010). ABA perception and signalling. Trends Plant Sci.

[24] Osakabe Y (2014). Response of plants to water stress. Front Plant Sci.

[25] Fan Q (2016). CmWRKY1 Enhances the Dehydration Tolerance of Chrysanthemum through the Regulation of ABA-Associated Genes. PLoS One.

[26] Yan HR (2014). The Cotton WRKY Transcription Factor GhWRKY17 Functions in Drought and Salt Stress in Transgenic *Nicotiana benthamiana* Through ABA Signaling and the Modulation of Reactive Oxygen Species Production. Plant Cell Physiol.

[27] Ren XZ (2010). ABO3, a WRKY transcription factor, mediates plant responses to abscisic acid and drought tolerance in Arabidopsis. Plant J.

[28] Saleem MF (2016). Understanding and mitigating the impacts of drought stress in cotton- a review. Pak J Agr Sci.

[29] Zhu XL (2018). The yield difference between wild-type cotton and transgenic cotton that expresses IPT depends on when water-deficit stress is applied. Sci Reprots.

[30] Zhou L (2014). Molecular characterization of 26 cotton WRKY genes that are expressed differentially in tissues and are induced in seedlings under high salinity and osmotic stress. Plant Cell Tiss Organ Cult.

[31] Chi Y (2013). Protein-protein interactions in the regulation of WRKY transcription factors. Mol Plant.

[32] Yehoram L (2010). Reduced expression of the v-SNAREs AtVAMP71/AtVAMP7C gene family in Arabidopsis reduces drought tolerance by suppression of abscisic acid-dependent stomatal closure. J Exp Bot..

[33] Xiong L (2006). Identification of drought tolerance determinants by genetic analysis of root response to drought stress and abscisic acid. Plant Physiol.

[34] Schroeder JI (2001). Guard cell signal transduction. Annu Rev Plant Physiol Plant Mol Biol.

[35] Eulgem T (2005). Regulation of the Arabidopsis defense transcriptome. Trends Plant Sci.

[36] Sun YD, Yu DQ (2015). Activated expression of AtWRKY53 negatively regulates drought tolerance by mediating stomatal movement. Plant Cell Rep.

[37] Jaffar MA (2016). Involvement of CmWRKY10 in Drought Tolerance of Chrysanthemum through the ABA-Signaling Pathway. Int J Mol Sci.

[38] Li FJ (2017). Regulation of cotton (*Gossypium hirsutum*) drought responses by mitogen-activated protein (MAP) kinase cascade-mediated phosphorylation of GhWRKY59. New Phytol.

[39] Jia HH (2015). GhWRKY68 Reduces Resistance to Salt and Drought in Transgenic *Nicotiana benthamiana*. PLoS One.

[40] Verslues PE (2006). Methods and concepts in quantifying resistance to drought, salt and freezing, abiotic stresses that affect plant water status. Plant J.

[41] Kim DJ, Ntui VO, Xiong LM (2016). Arabidopsis YAK1 regulates abscisic acid response and drought resistance. FEBS Lett.

[42] Li S, Assmann SM, Albert R (2006). Predicting essential components of signal transduction networks: a dynamic model of guard cell abscisic acid signaling. PLoS Biol.

[43] Finkelstein RR, Gampala SS, Rock CD (2002). Abscisic acid signaling in seeds and seedlings. Plant Cell.

[44] Li T (2016). A Dual-Function Transcription Factor, AtYY1, Is a Novel Negative Regulator of the Arabidopsis ABA Response Network. Mol Plant.

[45] Butt HI (2017). GaMYB85, an R2R3 MYB gene, in transgenic Arabidopsis plays an important role in drought tolerance. BMC Plant Biol.

[46] Luo X (2013). Expression of wild soybean WRKY20 in Arabidopsis enhances drought tolerance and regulates ABA signaling. J Exp Bot.

[47] Yamaguchi-Shinozaki K, Shinozaki K (1993). Characterization of the expression of a desiccation-responsive rd29 gene of *Arabidopsis thaliana* and analysis of its promoter in transgenic plants. Mol Gen Genet.

[48] Yamaguchi-Shinozaki K, Shinozaki K (1994). A novel cis-acting element in an Arabidopsis gene is involved in responsiveness to drought, low-temperature, or high-salt stress. Plant Cell.

[49] Yamaguchi-Shinozaki K, Shinozaki K (2006). Transcriptional regulatory networks in cellular responses and tolerance to dehydration and cold stresses. Annu Rev Plant Biol.

[50] Kariola T (2006). Early Responsive To Dehydration 15, a Negative Regulator of Abscisic Acid Responses in Arabidopsis. Plant Physiol.

[51] Xiao BZ (2009). Evaluation of Seven Function-Known Candidate Genes for their Effects on Improving Drought Resistance of Transgenic Rice under Field Conditions. Mol Plant.

[52] Lang V, Palva ET (1992). The expression of a rab-related gene, rab18, is induced by abscisic acid during the cold acclimation process of *Arabidopsis thalian*a (L.) Heynh. Plant Mol Biol.

[53] Babula-Skowrońska D (2015). Involvement of genes encoding ABI1 protein phosphatases in the response of *Brassica napus L*. to drought stress. Plant Mol Biol.

[54] Meyer K, Leube MP, Grill E (1994). A protein phosphatase 2C involved in ABA signal transduction in *Arabidopsis thaliana*. Science.

[55] Sheen J (1998). Mutational analysis of protein phosphatase 2C involved in abscisic acid signal transduction in higher plants. Proc Natl Acad Sci.

[56] Fujii H, Verslues PE, Zhu JK (2007). Identification of two protein kinases required for abscisic acid regulation of seed germination, root growth, and gene expression in Arabidopsis. Plant Cell.

[57] Qin LX (2014). Arabidopsis drought-induced protein Di19-3 participates in plant response to drought and high salinity stresses. Plant Mol Biol.

[58] Li XB (2005). The cotton ACTIN1 gene is functionally expressed in fibers and participates in fiber elongation. Plant Cell.

[59] Zhu SY (2007). Two calcium-dependent protein kinases, CPK4 and CPK11, regulate abscisic acid signal transduction in Arabidopsis. Plant Cell.

[60] Gong SY (2012). GhAGP31, a cotton non-classical arabinogalactan protein, is involved in response to cold stress during early seedling development. Plant Biol.

[61] Qin LX (2013). Cotton GalT1 encoding a putative glycosyltransferase is involved in regulation of cell wall pectin biosynthesis during plant development. PLoS One.

[62] Pei ZM (1997). Differential abscisic acid regulation of guard cell slow anion channels in Arabidopsis wild-type and abi1 and abi2 mutants. Plant Cell.

