# Supplementary material for: The cotton WRKY transcription factor (GhWRKY33) reduces transgenic Arabidopsis resistance to drought stress
Source: Sci Rep. 2019 Jan 24;9:724. doi: 10.1038/s41598-018-37035-2 (PMC6346051; doi:10.1038/s41598-018-37035-2)
Supplement: Supplementary file 1 — Supplementary information [file 41598_2018_37035_MOESM1_ESM.pdf]

The cotton WRKY transcription factor (GhWRKY33) reduces transgenic Arabidopsis resistance to drought stress

Na-Na Wang[1], Shang-Wei Xu[1], Yun-Lue Sun, Dong Liu, Li Zhou, Yang Li*, Xue-Bao Li*

Hubei Key Laboratory of Genetic Regulation and Integrative Biology, School of Life Sciences, Central China Normal University, Wuhan 430079, China

Supplementary data

Table S1. Oligonucleotide primers used in this study.

Primer	Sequence	Purpose
GhWRKY33 OE-F	5'-CTTGGATCCATGGAGCATAGATGGAACATG-3'	Gene cloning
GhWRKY33 OE-R	5'-CTTCTCGAGTTAGGAGGAGAAAAATCCTTGG-3'	Gene cloning
GhWRKY33GFP-F	5'-GCCTCTAGAATGGAGCATAGATGGAACATGG-3'	Gene cloning
GhWRKY33GFP-R	5'-CTTGGATCCGGAGGAGAAAAATCCTTGGGT-3'	Gene cloning
GhWRKY33 AD-F	5'-GGGGAATTCATGGAGCATAGATGGAACATGG-3'	Yeast one-hybrid
GhWRKY33 AD-R	5'-GGGGAGCTCTTAGGAGGAGAAAAATCCTTGGG-3'	Yeast one-hybrid
AtRD29A AD-F	5'-GGGGAGCTCGTATGATGCCTCTGTTTGTG-3'	Yeast one-hybrid
AtRD29A AD-R	5'-CTTGTCGACCAAACGGCACATCCTTCTC-3'	Yeast one-hybrid
AtERD15 AD-F	5'-GGGGAGCTCGGCTAGCGTTCAATACGATG-3'	Yeast one-hybrid
AtERD15 AD-R	5'-CTTGTCGACGGTGAGACACAAGTGGGTAG-3'	Yeast one-hybrid
AtRAB18 AD-F	5'-GGGGGTACCGTCACAAGCACTGAAGTCTG-3'	Yeast one-hybrid
AtRAB18 AD-R	5'-GGGGTCGACCGAATCTTCTACGGTTGGTC-3'	Yeast one-hybrid
AtABI1 AD-F	5'-CTTGAGCTCTCTACGTGTCGACCATCCAC-3'	Yeast one-hybrid
AtABI1 AD-R	5'-GGGGGTACCGACACTTGCTTCAGACTTCC-3'	Yeast one-hybrid
AtDREB2A AD-F	5'-CTTCCCGGGGTGTGCACGATCATTCGG-3'	Yeast one-hybrid
AtDREB2A AD-R	5'-GGGGTCGACGGTTTCCGGTGTACGTAC-3'	Yeast one-hybrid
AtSOS2 AD-F	5'-GGGCCCGGGGATGGGTTAATTGGATAAACC-3'	Yeast one-hybrid
AtSOS2 AD-R	5'-GGGGTCGACAGTCGTTCGGGTCAATTATCC-3'	Yeast one-hybrid
GhWRKY33 RT-F	5'-TGATTTCAGCCAACACTTCA-3'	QRT-PCR
GhWRKY33 RT-R	5'-ATCACTGGAGATGTCTTACA-3'	QRT-PCR
AtRD29A RT-F	5'-ATCACTTGGCTCCACTGTTGTTC-3'	QRT-PCR
AtRD29A RT-R	5'-ACAAAACACACATAAACATCCAAAGT-3'	QRT-PCR
AtDREB2A RT-F	5'-G AAGGAGCAAG GGATTGTAG-3'	QRT-PCR
AtDREB2A RT-R	5'-GTATGAACCGTTGGCAACACTG-3'	QRT-PCR
AtRAB18 RT-F	5'-CGAATGGCATCCTTTCTCAATC-3'	QRT-PCR
AtRAB18 RT-R	5'-GTCACCGAGAGTGCGGATATG-3'	QRT-PCR
AtSOS2 RT-F	5'-GGCTTGAAGAAAGTGAGTCTCG-3'	QRT-PCR
AtSOS2 RT-R	5'-GCTACATAGTTCGGAGTTCCACA-3'	QRT-PCR
AtABI1 RT-F	5'-AGATGGCAAGGAAGCGGATT-3'	QRT-PCR
AtABI1 RT-R	5'-CAACCACCACCACACTTATG-3'	QRT-PCR
AtERD15 RT-F	5'-TCAGCGAGGCTGGTGGATG-3'	QRT-PCR
AtERD15 RT-R	5'-TGAGAATGGCGATGGTATCAGGA-3	QRT-PCR
AtACT2 RT-F	5'- GAAATCACAGCACTTGCACC-3'	QRT-PCR
AtACT2 RT-R	5'- AAGCCTTTGATCTTGAGAGC-3'	QRT-PCR
